# Multi-locus sequence analysis reveals great genetic diversity among *Mycoplasma capricolum* subsp. *capripneumoniae* strains in Asia

**DOI:** 10.1186/s13567-022-01107-z

**Published:** 2022-11-14

**Authors:** Arooba Akhtar, Anne Boissière, Huafang Hao, Muhammad Saeed, Virginie Dupuy, Antoni Exbrayat, Farhan Anwar Khan, Yuefeng Chu, Lucía Manso-Silván

**Affiliations:** 1 College of Veterinary Sciences, Faculty of Animal Husbandry and Veterinary Sciences, The University of Agriculture, Peshawar, 25120 Khyber Pakhtunkhwa Pakistan; 2 State Key Laboratory of Veterinary Etiological Biology, College of Veterinary Medicine, Lanzhou University, Lanzhou Veterinary Research Institute, Chinese Academy of Agricultural Sciences, Lanzhou, China; 3 UMR ASTRE, CIRAD, 34398 Montpellier, France; 4 ASTRE, Univ. Montpellier, CIRAD, INRAE, Montpellier, France

**Keywords:** *Mycoplasma capricolum* subsp. *capripneumoniae*, contagious caprine pleuropneumonia, multi-locus sequence analysis, molecular epidemiology, Asia, Africa

## Abstract

Multi-Locus Sequence Analysis (MLSA) of *Mycoplasma capricolum* subsp. *capripneumoniae* (*Mccp*) strains from Asia revealed unforeseen diversity and a central position for genotyping groups representing strains from Central/East Asia, suggesting a possible origin of contagious caprine pleuropneumonia in this continent. A better assessment of the emergence, diversity and distribution of Mccp in Asia and Africa calls for renewed efforts to dramatically enlarge the sample of strains. Availability and affordability in the field, added to superior typeability (directly from poor samples) and high stability, discriminatory power and concordance with epidemiological and phylogenetic analyses, make MLSA an excellent tool for such investigations.

## Introduction, methods and results

Contagious caprine pleuropneumonia (CCPP) is a devastating disease affecting domestic goats and several wild ungulate species in arid and semiarid regions of Africa, Middle East and Asia, where goat rearing plays an essential role in food security and poverty alleviation [1]. Owing to its high contagiousness, morbidity and mortality, CCPP is included in the list of notifiable diseases of the World Organisation for Animal Health (WOAH, founded as OIE; [2]). Its etiologic agent, a fastidious bacterium known as *Mycoplasma capricolum* subsp. *capripneumoniae* (*Mccp*), is very rarely isolated and CCPP is hardly ever reported. As a consequence, the distribution, prevalence and impact of CCPP are not well established [3].

To improve our understanding on the epidemiology of CCPP, a molecular typing scheme based on the analysis of eight genetic markers, known as Multi-Locus Sequence Analysis (MLSA), was developed in 2011 [4]. This tool was extremely robust and allowed genotyping directly from infected tissues from which *Mccp* could not be isolated. The scheme was applied to 27 strains of diverse origins, resulting in the identification of two lineages and 5 groups, which were correlated to the geographic origin of the strains (with the remarkable exception of the Arabian Peninsula, where strains from 4 out of the 5 groups were found). Notably, the identification of a distinct Asian cluster represented by two recent strains from Tajikistan and China (sole representatives of Central and East Asia available at the time) indicated a local evolution of strains and excluded a recent introduction of CCPP in the continent.

Thanks to the democratisation of high throughput sequencing technologies more sophisticated *Mccp* strain genotyping methods have been developed, from a multi-gene scheme [5] to a whole-genome sequence (WGS) analysis pipeline [6], attaining optimum strain typing for molecular epidemiology studies and outbreak investigations. However, WGS-based genotyping is not available to diagnostic laboratories, particularly in the regions where CCPP is prevalent, and MLSA may still be a valuable alternative, especially when isolation cannot be achieved. Only a few *Mccp* isolates and WGS have been made available since the MLSA work of 2011 and subsequent reports relating to *Mccp* strains from wildlife in the United Arab Emirates [7, 8], but MLSA has been conducted following investigations of CCPP outbreaks in Tibetan wild ungulates first identified in 2012 [9] and, more recently, in Pakistani goats in 2019 [10]. The objective of our study was thus to explore the diversity of *Mccp* strains in Asia, by analysing new MLSA data from Pakistan and China, including strains originating from wildlife. This was also the opportunity to update the global *Mccp* MLSA, by including all the data generated since 2011, and to analyse its value and performance in comparison to subsequent typing techniques based on WGS data.

The 43 *Mccp* strains and/or corresponding genomic sequences analysed in this study are presented in Table 1, including 8 strains from wild ungulate species. Thirty-three of them were included in subsequent typing schemes [5, 6] and corresponding phylogenetic groups are presented when available. Sixteen new strains were added to 27 previously published [4]. MLSA data of 6 new strains were extracted from available WGS, while the remaining 10 were obtained by PCR amplification and sequencing of the corresponding eight loci as previously described [4], with the exception that Sanger sequencing was performed by Macrogen (South Korea), while Geneious 10.2.6 [11] was used for sequence assembly and alignment.

**Table 1:** **List of *Mccp*** **strains and genomes analysed in this study and corresponding MLSA types.**

Strain	Supplier	Reference	Year	^#^Geographic origin	Host	GenBank	MLSA	Group
97095-Tigray	NVI-E	[26]	1988	Ethiopia, Tigray	*Capra hircus*	ND	1-010	A
9277-PF1	VRA	[26]	< 1992	Sudan, NA	*Capra hircus*	ND	1-010	ND
99108-P1*	SVS	[26]	1999	Eritrea, Adi Keshi/Kenya, Tigray	*Capra hircus*	ND	1-010	A
04012 ^§^	AWWP	[20]	2004	Qatar, Al Shahaniya	*Capra aegagrus*	CP040917	1-010	A
13092	EAD	[7]	2013	UAE, Abu Dhabi	*Gazella marica*	ND	1-011	A
14001	EAD	[7]	2014	UAE, Abu Dhabi	*Oryx leucoryx*	ND	1-011	A
*16034*	EAD	[6]	2016	UAE, Al-Ain	*Oryx dammah*	ND	1-011	A
M74/93	NVI-S	[19]	1993	Uganda, Karamoja	*Ovis aries*	ND	1-020	ND
M79/93*	NVI-S	[19]	1993	Uganda, Karamoja	*Capra hircus*	ND	1-020	A
**149F09-SNC1**	VLA	[21]	2009	Mauritius, West	*Capra hircus*	ND	1-030	ND
*ILRI 181*	ILRI	[22]	2012	Kenya, Laikipia	*Capra hircus*	LN515399	1-030	A
*14020*	TVLA	[6]	2013	Tanzania, Manyara, Kiteto	*Capra hircus*	ND	1-040	A
8789	LRVZF	[27]	1987	Chad, Karal, Dandi	*Capra hircus*	ND	2-010	B
94156^§^	LRVZF	[26]	1994	Chad, N'Djamena	*Capra hircus*	CP041708	2-010	B
05021^§^	VRA	[4]	2004	Sudan, Darfour, Nyala	*Capra hircus*	CP041700	2-010	B
95043^§^	LABOCEL	[26]	1995	Niger, Goure	*Capra hircus*	CP041705	2-020	B
*M1601*	LVRI	[28, 29]	2007	China, Gansu	*Capra hircus*	CP017125	3-010	D
44F04	PVCRI	[30]	2004	Turkey, Thrace	*Capra hircus*	ND	3-020	C
09018	CIRAD	[31]	2009	Tajikistan, Rogun	*Capra hircus*	ND	3-020	ND
12002^§^	MoA-T	[5]	2011	Tajikistan, NA	*Capra hircus*	CP041702	3-020	C
C550/1^§^	CVRL	[26]	1991	UAE, Dubai	*Capra hircus*	CP041703	3-030	C
Gabes	CIRAD	[32]	1980	Tunisia, Gabes	*Capra hircus*	ND	4-010	E
Gabes/102p	CIRAD	[4]	1980	Tunisia, Gabes	*Capra hircus*	ND	4-010	E
LKD	CIRAD	[32]	1980	Tunisia, Kebili Douz	*Capra hircus*	ND	4-010	E
9081-487P	MAF-O	[26]	1990	Oman, NA	*Capra hircus*	ND	4-010	E
07033-033C1^§^	FU	[33]	2007	Turkey, Elazig	*Capra hircus*	CP041712	4-010	E
7/2^§^	MRI	[34]	1988	Oman, NA / Turkey, NA	*Capra hircus*	CP041701	4-020	E
97097-Erer^§^	NVI-E	[26]	1997	Ethiopia, Erer	*Capra hircus*	CP041706	5-010	F
AMRC-C758^§^	AU	[35]	1981	Sudan, NA	*Capra hircus*	CP041711	5-020	F
Yatta B^§^	NVI- S	[26]	< 1997	Kenya, Yatta	*Capra hircus*	CP041707	5-020	F
F38^§^	CIRAD	[12, 22]	1976	Kenya, NA	*Capra hircus*	LN515398	5-030	F
94029-C5^§^	AVS	[26]	1994	Oman, NA	*Capra hircus*	CP041709	5-040	F
91039-C3^§^	NVI-E	[36]	1991	Ethiopia, Awash	*Capra hircus*	CP041710	5-050	F
9231-Abomsa^§^	CIRAD	[36, 37]	1982	Ethiopia, Gojjam	*Capra hircus*	LM995445	5-060	F
92138-CLP1	NVI-E	[26]	1992	Ethiopia, Bishoftu	*Capra hircus*	ND	5-060	F
**1303-SF**	LVRI	NA	2013	China, Tibet, Nagqu	*Ovis aries*	ND	6-010	ND
SD3	HVRI	[38]	2006	China, Shandong	*Capra hircus*	ND	6-020	ND
*87001*	HVRI	[25, 39]	1958	China, Shandong	*Capra hircus*	CP006959	6-030	G
**1209LFT**	LVRI	NA	2012	China, Tibet, Nagqu	*Pantholops hodgsonii*	ND	6-040	ND
***1411LFT1***	LVRI	NA	2014	China, Tibet, Nagqu	*Pantholops hodgsonii*	CP101367	6-040	ND
***zly1402F***	LVRI	NA	2014	China, Tibet, Nagqu	*Pantholops hodgsonii*	ND	6-040	ND
*zly1309F*	LVRI	[40]	2013	China, Tibet, Nagqu	*Pantholops hodgsonii*	CP019061	6-050	H
Gilgit	UoA-P	[10]	2019	Pakistan, Baltistan, Gilgit	*Capra hircus*	ND	7-010	ND

Out of 43 strains listed 39 were used for diversity analysis, with additional strains/passages originating from the same or consecutive outbreaks (framed) used for stability analysis. New strains not included in 2011 [4] are underlined and those not previously genotyped are double underlined. MLSA data from 36 strains was obtained by locus amplification and sequencing, of which 5 (in bold) directly from non-viable samples. Strains for which MLSA data were exclusively extracted from genomic data are italicised. Corresponding whole genome sequence typing groups according to [5] and [6] are provided when available.

*AU* Aarhus University, Denmark,* AVS* Agriculture and Veterinary Services, Oman,* AWWP* Al Wabra Wildlife Preservation, Qatar, *CIRAD* Centre de coopération international en recherche agronomique pour le développement, France, *CVRL* Central Veterinary Research Laboratory, UAE, *EAD* Environment Agency, Abu Dhabi, UAE, *FU* Firat University, Turkey, *HVRI* Harbin Veterinary Research Institute, China, *ILRI* International Livestock Research Institute, Kenya, *LABOCEL* Laboratoire Central de l’Elevage de Niamey, Niger, *LRVZF* Laboratoire de Recherches Vétérinaires et Zootechniques de Farcha, Chad, *LVRI* Lanzhou Veterinary Research Institute, China, *MAF-O* Ministry of Agriculture and Fisheries, Oman, *MRI* Moredun Research Institute, UK, *MoA-T* Ministry of Agriculture, Tajikistan, *NA* Non-Available, *ND* Not Determined, *NVI-E* National Veterinary Institute, Ethiopia, *NVI-S* National Veterinary Institute, Sweden, *PVCRI* Pendik Veterinary Control and Research Institute, Turkey, *SVS* Senhit Veterinary Service, Eritrea, *TVLA* Tanzania Vet Lab Agency, *UAE* United Arab Emirates, *UoA-P* University of Agriculture, Pakistan, *VLA* Veterinary Laboratory Agency, Weybridge, UK,*VRA* Veterinary Research Administration, Sudan.

^#^place of isolation/previous location of the animals.

^*^could not be differentiated by large-scale genotyping [5].

^§^MLSA data obtained both by PCR and sequencing and by extraction from genomic data.

The sequences of epidemiologically-related strains collected in nearby locations during CCPP epizootics in Uganda, Tunisia and Tibet or obtained by in vitro passage (Table 1) were identical, showing that the molecular markers were stable and there were no laboratory-introduced variations. Furthermore, MLSA results obtained by locus amplification and sequencing versus extraction from WGS data (for 15 strains analysed by PCR and sequencing with WGS available in GenBank, Table 1) were also identical. The only exception was strain F38, for which a single nucleotide polymorphism (SNP) in the H2 locus differentiated MLSA sequences obtained by the two methods. However, since two different laboratory stocks of this strain were used for PCR and sequencing (CIRAD) and WGS (NCTC 10192 T), this SNP may result from divergent evolution undergone by the two laboratory stocks from the original 1974 isolate [12]. When the scheme was applied to the remaining 39 “unrelated” strains in Table 1, 24 sequence types (ST) (9 new) were discriminated based on 68 polymorphic positions (16 new), which are shown in Table 2, with locus sequences from *Mccp* type strain F38 serving as reference. This resulted in a Simpson’s index of diversity of 0.970 (0.953–0.987), which expresses the probability of two unrelated strains being characterised as the same type [13, 14]. All the strains could be discriminated individually by WGS analysis [6] and all but two of those analysed by Dupuy et al. [5] provided distinct genotypes (Table 1). However, these two isolates were actually discriminated by MLSA, which allowed typing of non-viable strains (*n* = 6, Table 1) with no added difficulty or cost.

**Table 2:** **Sequence polymorphisms found among the** ***Mccp*** **strains analysed.**

Position^#^	**Locus 1**									**Locus 3**								**Locus 11**								**Locus 12 **				**Locus 15**				**Locus 17**
	56	74	258	320	417	433	441	460	461	54	222	244	528	534	546	579	597	7	54	247	391	566	586	605	625	31	533	542	567	11	406	451	649	18
									462					535										606										
F38	G	A	C	G*	G*	A	A	T*	-*	C	G	C	A	-	T	G	G*	C*	C	G*	G	C	G	-	C	A	A	G	G	T	C	T	T	G
1-010	A	G	G	.	.	.	.	.	.	.	.	.	C	.	C	T	.	.	T	.	A	.	.	.	.	G	G	A	A	G	T	.	C	A
1-011	A	G	G	.	.	.	.	.	.	.	.	.	C	.	C	T	A	.	T	.	A	.	.	.	.	G	G	A	A	G	T	.	C	A
1-020	A	G	G	.	.	.	G	.	.	.	.	.	C	.	C	T	.	.	T	.	A	.	.	.	.	G	G	A	A	G	T	.	C	A
1-030	A	G	G	.	.	.	G	.	.	.	.	.	C	.	C	T	.	.	T	.	A	.	.	.	.	G	G	A	A	G	T	.	C	A
1-040	A	G	G	A	.	.	G	.	.	.	.	.	C	.	C	T	.	.	T	.	A	.	.	.	.	G	G	A	A	G	T	.	C	A
2-010	A	G	G	.	.	T	.	.	.	.	.	.	C	T	C	T	.	.	T	.	A	T	.	.	.	G	G	A	A	G	.	C	C	A
2-020	A	G	G	.	.	T	.	.	.	.	.	.	C	T	C	T	.	.	T	.	A	T	.	.	.	G	G	A	A	G	.	C	C	A
3-010	.	G	.	.	.	.	.	.	.	.	.	T	C	.	C	.	.	.	.	.	.	.	.	.	.	.	.	A	A	G	.	.	.	.
3-020	.	G	.	.	.	.	.	.	.	.	.	.	C	.	C	.	.	.	.	.	.	.	.	.	.	.	.	A	A	G	.	.	.	.
3-030	.	G	.	.	.	.	.	.	.	.	A	.	C	.	C	.	.	.	.	.	.	.	.	.	.	.	.	A	A	G	.	.	.	.
4-010	.	G	.	.	.	.	.	.	.	.	.	.	C	.	C	.	.	.	.	.	.	.	.	.	.	.	.	.	A	.	.	.	.	.
4-020	.	G	.	.	.	.	.	.	.	.	.	.	C	.	C	.	.	.	.	.	.	.	A	.	.	.	.	.	A	.	.	.	.	.
5-010	.	.	.	.	.	.	.	.	.	.	.	.	.	.	.	.	.	.	.	.	.	.	.	.	.	.	.	.	A	.	.	.	.	.
5-020	.	.	.	.	.	.	.	.	.	.	.	.	.	.	.	.	.	.	.	.	.	.	.	.	.	.	.	.	.	.	.	.	.	.
5-030	.	.	.	.	.	.	.	.	.	.	.	.	.	.	.	.	.	.	.	.	.	.	.	.	.	.	.	.	.	.	.	.	.	.
5-040	.	.	.	.	.	.	.	.	.	T	.	.	.	.	.	.	.	.	.	.	.	.	.	.	.	.	.	.	.	.	.	.	.	.
5-050	.	.	.	.	.	.	.	.	.	T	.	.	.	.	.	.	.	.	.	.	.	.	.	A	.	.	.	.	.	.	.	.	.	.
5-060	.	.	.	.	.	.	.	.	.	T	.	.	.	.	.	.	.	.	.	.	.	.	.	A	T	.	.	.	.	.	.	.	.	.
6-010	.	G	.	.	.	.	.	.	T	.	.	.	C	.	C	.	.	T	.	A	A	.	.	.	.	.	.	A	A	G	.	.	.	.
6-020	.	G	.	.	.	.	.	.	.	.	.	.	C	.	C	.	.	T	.	A	A	.	.	.	.	.	.	A	A	G	.	.	.	.
6-030	.	G	.	.	.	.	.	-	.	.	.	.	C	.	C	.	.	T	.	A	A	.	.	.	.	.	.	A	A	G	.	.	.	.
6-040	.	G	.	.	A	.	.	.	T	.	.	.	C	.	C	.	.	T	.	A	A	.	.	.	.	.	.	A	A	G	.	.	.	.
6-050	.	G	.	.	A	.	.	.	.	.	.	.	C	.	C	.	.	T	.	A	A	.	.	.	.	.	.	A	A	G	.	.	.	.
7-010	.	.	.	.	.	.	.	.	.	.	.	.	.	.	.	.	.	.	.	.	.	.	.	A	T	.	.	.	.	G	.	.	.	.

Position^#^	**Locus 17**								**LOCUS 20**								**Locus H2**																	
	28	104	132	157	256	468	529	639	29	112	119	317	506	575	658	681	35	97	116	149	374	763	1024	1111	1149	1259	1341	1409	1430	1454	1778	1940	1974	2103
				158														98								1260								
F38	C	C	G*	-	A	A	G	T	T	A	G*	G	C	T	C	T	G	-*	T*	T*	C	G	G	C*	T	-*	G*	G*	G	G	C	G	T	C
1-010	.	T	.	.	G	G	A	G	.	G	.	A	.	C	T	C	A	.	.	.	.	.	.	.	.	.	.	.	.	.	.	.	C	.
1-011	.	T	.	.	G	G	A	G	.	G	.	A	.	C	T	C	A	.	.	.	.	.	.	.	.	.	.	.	.	.	.	.	C	.
1-020	.	T	.	.	G	G	A	G	.	G	.	A	.	C	T	C	A	.	.	.	.	.	.	.	.	.	.	.	.	.	.	.	C	.
1-030	.	T	.	.	G	G	A	G	.	G	.	A	.	C	T	C	A	.	.	.	.	.	.	.	.	.	.	A	.	.	.	.	C	.
1-040	.	T	A	.	G	G	A	G	.	G	.	A	.	C	T	C	A	.	.	.	.	.	.	.	.	.	.	.	.	.	.	.	C	.
2-010	.	T	.	T	G	.	A	G	.	G	.	A	.	C	T	C	.	.	.	.	.	.	.	.	C	.	.	.	.	.	.	A	C	G
2-020	.	T	.	.	G	.	A	G	C	G	.	A	.	C	T	C	.	.	.	.	.	.	.	.	C	.	.	.	A	.	.	A	C	G
3-010	T	.	.	.	G	.	.	G	.	.	.	.	.	C	T	C	.	.	.	.	.	.	.	.	.	.	.	.	.	.	.	.	C	.
3-020	.	.	.	.	G	.	.	G	.	.	.	.	.	C	T	C	.	.	.	.	.	.	T	-	-	.	-	-	-	-	-	-	-	.
3-030	.	.	.	.	G	.	.	G	.	.	.	.	.	C	T	C	.	.	.	.	.	.	T	-	-	.	-	-	-	-	-	-	-	.
4-010	.	.	.	.	.	.	.	.	.	.	.	.	.	.	T	C	.	.	.	.	T	.	.	.	.	.	.	.	.	A	T	.	C	.
4-020	.	.	.	.	.	.	.	.	.	.	.	.	.	.	T	C	.	.	.	.	T	.	.	.	.	.	.	.	.	A	T	.	C	.
5-010	.	.	.	.	.	.	.	.	.	.	.	.	.	.	T	.	.	.	.	.	.	A	.	.	.	.	.	.	.	.	.	.	C	.
5-020	.	.	.	.	.	.	.	.	.	.	.	.	.	.	.	.	.	.	.	.	.	.	.	.	.	.	.	.	.	.	.	.	C	.
5-030	.	.	.	.	.	.	.	.	.	.	.	.	.	.	.	.	.	.	.	.	.	.	.	.	.	.	.	.	.	.	.	.	.	.
5-040	.	.	.	.	.	.	.	.	.	.	.	.	A	.	T	.	.	.	.	.	.	.	.	.	.	.	.	.	.	.	.	.	C	.
5-050	.	.	.	.	.	.	.	.	.	.	.	.	A	.	T	.	.	.	.	.	.	.	.	.	.	.	.	.	.	.	.	.	C	.
5-060	.	.	.	.	.	.	.	.	.	.	.	.	A	.	T	.	.	.	.	.	.	.	.	.	.	.	.	.	.	.	.	.	C	.
6-010	.	.	.	.	G	.	.	G	.	.	A	.	.	C	T	C	.	.	.	.	.	.	.	G	.	.	.	.	.	.	.	.	C	.
6-020	.	.	.	.	G	.	.	G	.	.	.	.	.	C	T	C	.	.	.	G	.	.	.	.	.	.	A	.	.	.	.	.	C	.
6-030	.	.	.	.	G	.	.	G	.	.	.	.	.	C	T	C	.	.	.	G	.	.	.	.	.	.	A	.	.	.	.	.	C	.
6-040	.	.	.	.	G	.	.	G	.	.	.	.	.	C	T	C	.	A	.	.	.	.	.	.	.	A	A	.	.	.	.	.	C	.
6-050	.	.	.	.	G	.	.	G	.	.	.	.	.	C	T	C	.	A	-	.	.	.	.	.	.	A	A	.	.	.	.	.	C	.
7-010	.	.	.	.	G	.	.	G	.	.	.	.	.	C	T	C	.	.	.	.	.	.	.	.	.	.	.	.	.	.	.	.	C	.

The polymorphisms found in the eight MLSA loci for each sequence type are shown, with new polymorphisms identified in this study indicated with an asterisk. The GenBank accession numbers corresponding to the eight F38 locus sequences used as reference are as follows: Loc-01: HQ864744, Loc-03: HQ864761, Loc-11: HQ864776, Loc-12: HQ864786, Loc-15: HQ864807, Loc-17: HQ864814 and Loc-20: HQ864737, Loc-H2: AF162991.

^#^Position as on Mccp F38 sequences (corresponding to sequence type 5-030).

**Figure 1:**
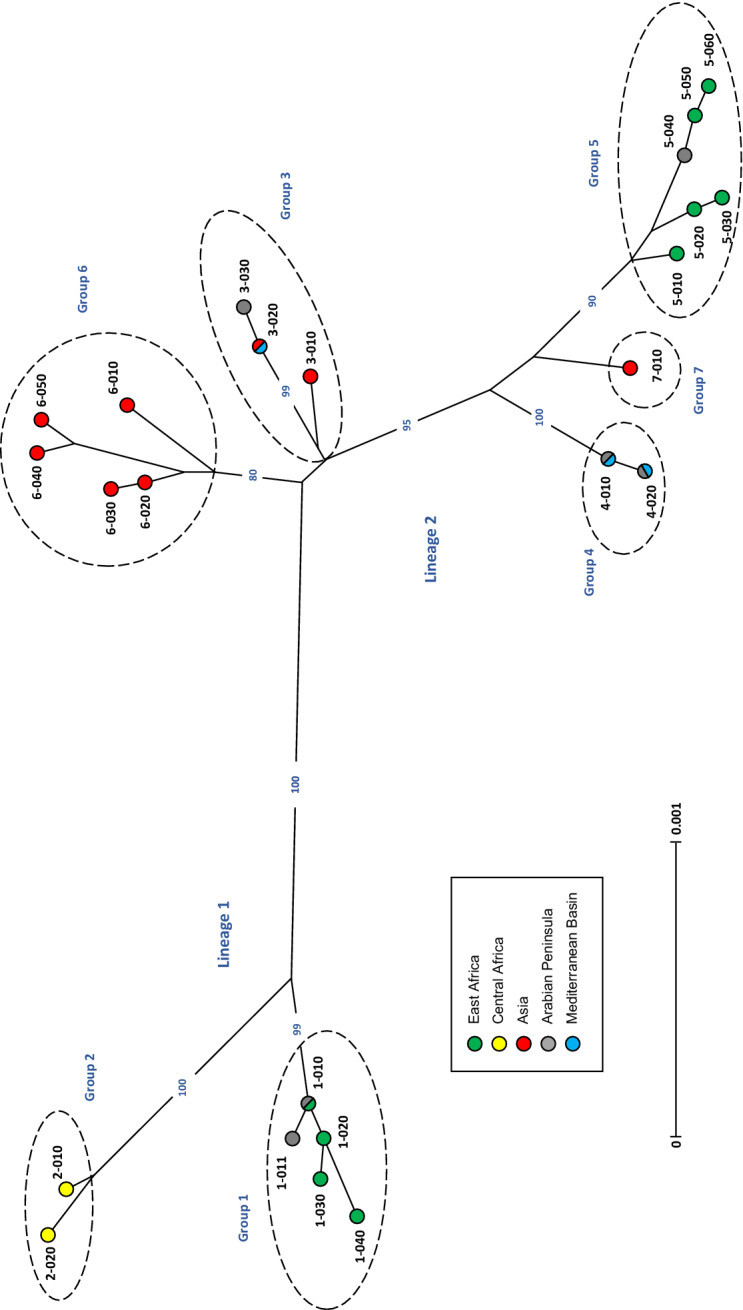
**Tree derived from distance analysis of the eight concatenated MLSA loci.** Neighbour-joining tree (DARwin 6) based on the analysis of a 6753 bp-sequence resulting from concatenation of the eight MLSA loci corresponding to the 24 sequence types identified among 43 (39 unrelated) strains (Table 1). Genotypes are assigned colour categories according to their geographical origin. Bootstrap percentage values were calculated from 1000 resamples and values over 80% are shown. The scale bar shows the equivalent distance to 1 substitution per 1000 nucleotide positions.

**Figure 2:**
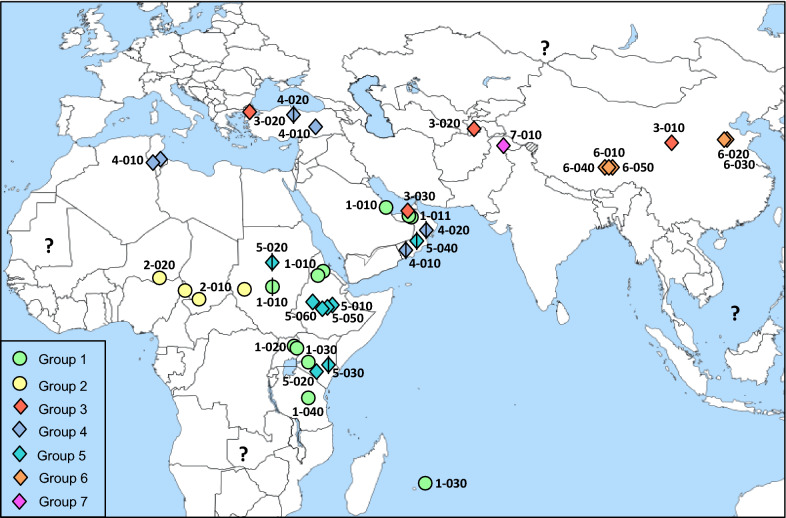
**Geographic distribution of the strains analysed in this study.** Each strain is represented by a symbol corresponding to its MLSA group (with circles and diamonds of various colours representing lineage I and II respectively) and its specific sequence type is indicated at the proximity. Strains for which the precise location was not known are indicated by barred symbols, placed arbitrarily in the country of origin. Question marks indicate areas from where no recent data is available.

A robust tree (Figure 1) was obtained by distance analysis of MLSA data using DARwin 6 [15] as previously described. Seven genotyping groups were identified, distributed in the two lineages previously described. Pre-existing MLSA groups 1–5 were unchanged, with the exception of several additional ST identified in group 1, corresponding to East African and Emirati strains originating from domestic goat and wild ungulates respectively. The remaining new ST identified in this work corresponded to Asian strains and were clustered in two additional groups, positioned within lineage II. A highly variable cluster located near the centre of the tree and represented by Chinese strains from Shandong and Tibet was designated group 6, whereas the Pakistani strain constituted the single representative of group 7. All Asian strains (disregarding those originating from the Middle East) were found spread among three clusters (groups 3, 6 and 7) within lineage II, together with group 4 (represented by strains from North Africa, the Arabian Peninsula and Turkey) and group 5 (comprising mainly East African strains). As shown in Figure 2, a generally good correlation between ST and geographic origin was retained, with the exception of the Arabian Peninsula, where animals from diverse origins are imported every year, particularly at the occasion of Muslim feasts [4]. A similar situation was now observed in Turkey, since strains from Thrace and Elazig (East Turkey) were positioned in groups 3 and 4 respectively.

## Discussion

The relevance of the MLSA scheme for *Mccp* genotyping and epidemiology analyses is unquestionable, particularly when we consider its accessibility, affordability, ease of use and superior typeability, allowing direct genotyping from poor samples. Furthermore, its stability, regardless the method used to obtain the data, was remarkable and MLSA clustering was highly congruent with both epidemiological and phylogenetic analyses [5, 6]. Finally, its high discriminatory power was compatible with epidemiological investigations.

Analysis of new strains from Pakistan and China allowed a better representation of the spread of CCPP in Asia (Figure 2) and revealed unpredicted diversity in this continent (Figure 1). The Pakistani strain was the sole representative of a new cluster (group 7), the diversity and distribution of which remain to be disclosed. This was unfortunately the only strain available from South Asia, where the occurrence of CCPP was documented as early as 1914 [16] and where CCPP is known to be prevalent [10, 17, 18]. The new Chinese strains constituted a distinct cluster (group 6), separate from previously described Tajik and Chinese strains (group 3). A strain from Tibetan sheep collected in the Nagqu region of Tibet (Table 1), where devastating CCPP outbreaks have been reported in both domestic goat and antelope since 2012 [9],was placed at the base of this group. This strain was more closely related to strains from domestic goats collected at Shandong than to strains from Tibetan antelope collected at Nagqu. This may be explained by the wide area of distribution of domestic and wild ungulate species across the Qinghai-Tibetan plateau and its peripheral mountains. It was assumed that Tibetan antelopes were infected due to close contact with domestic goats, which are progressively invading their habitat [9]. Furthermore, strains from CCPP outbreaks affecting domestic sheep in Uganda [19] and four different wild ungulate species in the Middle East [7, 8, 20] (Table 1), were placed in group 1, very distant to those from Tibetan wildlife, and shared or were closely related to ST from goat isolates, indicating that the same strains can affect a wide variety of species. Again, the assumption was that domestic goats were the source of the infection in sheep and wildlife, though direct Mccp transmission among infected wild ungulates of different species has been demonstrated, at least in captivity [8, 20].

Analysis of new Emirati strains from wildlife and additional strains from East Africa resulted in the identification of three new ST in group 1, revealing greater diversity for this cluster, which is spreading in the region. The strain introduced in Mauritius in 2009 [21], shared ST with a highly virulent Kenyan isolate from 2012 [22, 23] and was closely related to a strain that was responsible for CCPP outbreaks across Tanzania in 2013 [6]. The relatively low diversity of this group, and generally of lineage I compared to lineage II, deserves further investigation. Similarly, the presence in East Africa of two distant genotyping groups (one from each lineage), suggesting two different introductions of CCPP in this region, needs to be elucidated for a better understanding of the origin and evolution of CCPP in Africa.

CCPP was suspected in India and China since the beginning of the twentieth century [16, 24], but its presence in Asia was only confirmed in 2007 [25]. Already in 2011, MLSA genotyping suggested that CCPP was present for a long time in Asia [4], which was substantiated by subsequent large-scale genomic analyses [5, 6]. The great genetic diversity observed here among Asian *Mccp* strains in spite of the limited number of samples analysed, together with the position of MLSA groups 3 and 6 (represented by Central and East Asian strains) at the centre of the tree, point towards a possible origin of CCPP in Asia. Again, the scarcity of *Mccp* strains hampers a precise determination of the emergence, diversity and distribution of *Mccp*.

A better assessment of the molecular evolution and epidemiology of CCPP in Asia and Africa calls for renewed efforts to dramatically enlarge the sample of strains from diverse origins representing the real distribution of CCPP, which is yet to be established (Figure 2). MLSA can be an excellent tool to do this, provided CCPP cases are investigated, since these analyses can be achieved from simple samples such as dried filter paper imbedded in infected material, which can be easily stored and shipped at room temperature.

## Data Availability

The locus sequences corresponding to new MLSA sequence types 1–011, 1–040, 6–010, 6–020 and 7–010 obtained in this study, for which no representative sequences are available, were submitted to GenBank (accession numbers: OP076701–OP076740).
